# EMMPRIN Expression in Oral Squamous Cell Carcinomas: Correlation with Tumor Proliferation and Patient Survival

**DOI:** 10.1155/2014/905680

**Published:** 2014-05-21

**Authors:** Luís Silva Monteiro, Maria Leonor Delgado, Sara Ricardo, Fernanda Garcez, Barbas do Amaral, José Júlio Pacheco, Carlos Lopes, Hassan Bousbaa

**Affiliations:** ^1^Molecular Oncology Group, Institute of Research and Advanced Training in Health Sciences and Technologies (IINFACTS), Higher Institute of Health Sciences, CESPU, 4585-116 Paredes, Portugal; ^2^Medicine and Oral Surgery Department, Higher Institute of Health Sciences, 4585-116 Paredes, Portugal; ^3^Pathology Department, Higher School of Health of Vale do Sousa, 4585-116 Paredes, Portugal; ^4^Differentiation and Cancer Group, Institute of Molecular Pathology and Immunology of the University of Porto, (IPATIMUP), 4200-465 Porto, Portugal; ^5^Stomatology Department, Santo António Hospital, Oporto Hospitalar Centre, 4099-001 Porto, Portugal; ^6^Molecular Pathology and Immunology Department, Institute of Biomedical Sciences Abel Salazar (ICBAS), Porto University, 4050-313 Porto, Portugal; ^7^Centro de Química Medicinal da Universidade do Porto (CEQUIMED-UP), Rua de Jorge Viterbo Ferreira 228, 4050-313 Porto, Portugal; ^8^Centro Interdisciplinar de Investigação Marinha e Ambiental (CIIMAR/CIMAR), Universidade do Porto, Rua dos Bragas 289, 4050-123 Porto, Portugal

## Abstract

The aim of our study was to explore the clinicopathological and prognostic significance of extracellular matrix metalloproteinase inducer (EMMPRIN) expression in oral squamous cell carcinomas (OSCC), and its relation with the proliferative tumor status of OSCC. We examined EMMPRIN and Ki-67 proteins expression by immunohistochemistry in 74 cases with OSCC. Statistical analysis was conducted to examine their clinicopathological and prognostic significance in OSCC. EMMPRIN membrane expression was observed in all cases, with both membrane and cytoplasmic tumor expression in 61 cases (82.4%). EMMPRIN overexpression was observed in 56 cases (75.7%). Moderately or poorly differentiated tumors showed EMMPRIN overexpression more frequently than well-differentiated tumors (*P* = 0.002). Overexpression of EMMPRIN was correlated with high Ki-67 expression (*P* = 0.004). In the multivariate analysis, EMMPRIN overexpression reveals an adverse independent prognostic value for cancer-specific survival (CSS) (*P* = 0.034). Our results reveal that EMMPRIN protein is overexpressed in more than two-thirds of OSCC cases, especially in high proliferative and less differentiated tumors. The independent value of EMMPRIN overexpression in CSS suggests that this protein could be used as an important biological prognostic marker for patients with OSCC. Moreover, the high expression of EMMPRIN makes it a possible therapeutic target in OSCC patients.

## 1. Introduction

Oral cancer remains a major public health problem with almost 300,000 new cases worldwide [1, 2]. New insights in cancer diagnosis and therapy have not changed significantly the survival rate for oral cancer (around 50%) during the last decades [1].

Oral tumorigenesis is a multistep process caused by accumulation of multiple genetic and epigenetic alterations [3]. The comprehension of the molecular pathways involved in this process may originate special biological markers able to differentiate tumors with a more or less aggressive behavior. These markers may contribute to identify and stratify patients with greater precision to the most appropriate treatment plan. Moreover, these molecules could become molecular therapeutic targets.

Extracellular matrix metalloproteinase inducer (EMMPRIN), also known as CD147, Basignin, M6, Neurothelin, or gp42, is a highly glycosylated transmembrane protein, member of the immunoglobulin superfamily of receptors, discovered by its capacity of inducing the expression of matrix metalloproteinases [4]. It is present in epithelial cells, neuronal or nerve cells, myocardial cells, lymphoid cells, or germ cells and has an important role in several biological processes such as fetal development, retinal function, development of the nervous system and thymic T cell development [5]. EMMPRIN is expressed in several cancers including head and neck squamous-cell carcinomas, pancreatic adenocarcinomas, kidney chromophobic carcinomas, hepatocellular carcinomas, medullary breast adenocarcinomas, cervix carcinomas, and glioblastomas [5]. EMMPRIN contributes to cell adhesion modulation, tumor growth, invasion, and angiogenesis [4–7] probably due to its association with several proteins implicated in different signaling pathways such as matrix metalloproteinases, ErbB, MAPK cascade proteins, monocarboxylate transporters (MCT), integrins, caveolin-1 (Cav-1), Tenascin (TN)-C, vascular endothelial growth factor (VEGF), urokinase-type plasminogen activator (uPA), and cyclophilins (Cyp) [4, 6, 8, 9].

Previous reports have shown that EMMPRIN expression is associated with a high tumor aggressive behavior and with poor prognosis in several tumors [10–19]. However, in OSCC the prognostic significance of EMMPRIN is poorly studied. Moreover, the relation of this glycoprotein with the proliferative tumor capacity in patients with OSCC has not been reported.

We aimed in this study to evaluate the expression of EMMPRIN in patients with OSCC and investigate the association of this glycoprotein with clinicopathological, tumor proliferation, and prognosis variables.

## 2. Material and Methods

### 2.1. Patient Recruitment

This retrospective study included patients with newly diagnosed and consecutively treated primary OSCC at the Hospital de Santo António (HSA), Porto, Portugal, between 2000 and 2006. The study was approved by the institutional review board of the hospital. From patient's records, we obtained patient's age, gender, tumor location, tumor stage (I–IV), primary treatment, histological type, tumor grade, surgical margin status, and follow-up information.

Patients were excluded if they lacked clinical and follow-up information or if their paraffin blocks lacked sufficient tumor tissue leaving 74 patients for this study, 55 men and 19 women, with a mean age of 62.3 ± 15.3 years (range from 25 to 96 years).  Table 1 lists the clinicopathological features of these patients.

Tumor stage was classified according to the 7th edition of the classification of malignant tumors of American Joint Committee on Cancer [20]. For all tumors, 3 *μ*m sections were cut and stained with haematoxylin-eosin (HE) to confirm the initial diagnosis. Tumor grade was reclassified following the WHO classification (2005) into well-differentiated (G1), moderately differentiated (G2), and poorly differentiated (G3) OSCC [21]. Inspection for possible presence of tumor lymphatic invasion and perineural permeation reported as present or absent was performed on each sample.

### 2.2. Tissue Microarray (TMA) Construction

Immunohistochemistry was performed on tumor tissues using TMA technology designed and constructed according to rules previously described [22]. Briefly, representative tumor areas were selected on haematoxylin and eosin-stained sections and marked on paraffin blocks, avoiding necrosis and keratin areas. Three cylindrical tissue cores (2 mm in diameter) were obtained from each selected specimen and transferred to a recipient paraffin block, using a microarray instrument (TMA Builder, Histopathology Ltd., Hungary). From each TMA block, 3 *μ*m sections were cut and processed for immunohistochemistry.

### 2.3. Immunohistochemistry

TMA slides were deparaffinised in xylene, dehydrated in an ethanol series, and rinsed in distilled water. Epitope retrieval treatment was performed using 0.01 M citrate buffer (pH 6.0) for CD147 and 0.01 M trisethylenediaminetetraacetic acid (EDTA) buffer (pH 9.0) for Ki-67 at high temperature (98°C water bath during 30 minutes). After blocking endogenous peroxidase with methanol containing 0.3% hydrogen peroxide (H_2_O_2_) for 5 min, sections were incubated with a blocking solution made of 0.4% casein in trisbuffered saline (TBS) to reduce nonspecific binding. TMA slides were incubated with the primary monoclonal antibody (anti-CD147, clone AB1843, Novocastra, Newcastle upon Tyne, UK, diluted at 1 : 30; and anti-Ki-67, clone MIB1, Dako, Glostrup, Denmark, diluted at 1 : 10) during 60 minutes at room temperature. Then the slides were washed in TBS, followed by incubation with standard peroxidase-labelled dextran polymer for visualization with diaminobenzidine as chromogen (NovoLink Polymer Detection System, Novocastra, Leica Biosystems Newcastle Ltd.), according to the manufacturer's instructions. TMA tissue sections were lightly counterstained with Mayer haematoxylin for 2 min and cover-slipped. Positive (skin and oral mucosa) and negative (omission of primary antibody) controls were used in each staining run.

### 2.4. Evaluation of Immunohistochemical Expression

All samples were evaluated by two authors blinded to clinicopathological characteristics. The discordant cases were reviewed under a multihead microscope to achieve a consensus. We used the higher score out of at least 2 of the 3 cores examined per case. EMMPRIN staining was evaluated on the basis of extent and intensity immunolabeling of tumor cells. The intensity of staining was scored as 0 (absent), 1 (weak), 2 (moderate), and 3 (strong). The extent of membrane tumor cells staining was semiquantitatively evaluated as 0 (no labelling or labelling in <10% of tumor cells); 1 (labelling in 10% to 24% of tumor cells); 2 (labelling in 25% to 49% of tumor cells); 3 (labelling in 50% to 74% of tumor cells); and 4 (labelling in 75% or more of tumor cells). The sum of the intensity and extent scores was used as the final score (0–7). Tissues having a final score of 0-1 were considered negatives. Final scores of 2-3, 4-5, and 6-7 were considered 1+, 2+, and 3+, respectively. For data analysis, score 3+ was defined as EMMPRIN overexpression [12].

For Ki-67 evaluation, we considered the percentage of nuclear staining for scoring proliferative status. We classified tumors into two groups: low proliferative tumor (labelling from 0 to 49% of tumor cells) and high proliferative tumor (labelling in 50% or more of tumor cells) [23].

### 2.5. Statistical Analysis

Statistical analysis was carried out using IBM SPSS Statistics version 21.0 software (IBM Corporation, NY, US). The associations between categorical variables were evaluated by chi-square tests. Correlation between EMMPRIN and Ki-67 was measured by Spearman's correlation coefficient. Cancer-specific survival (CSS) was defined as the time interval (months) between primary treatment and death from oral cancer or last follow-up. Recurrence-free survival (RFS) was defined as the time interval (months) between primary treatment and the first recurrence (whether local, regional, or distant). The Kaplan-Meier method was used to plot survival curves and their prognostic effect was tested using the log-rank test. Variables with significant effects in the univariate analyses were entered into Cox proportional hazards model to investigate the independent effects of these variables. Differences were considered statistically significant at *P* < 0.05.

## 3. Results

### 3.1. EMMPRIN Expression

**Figure 1:**
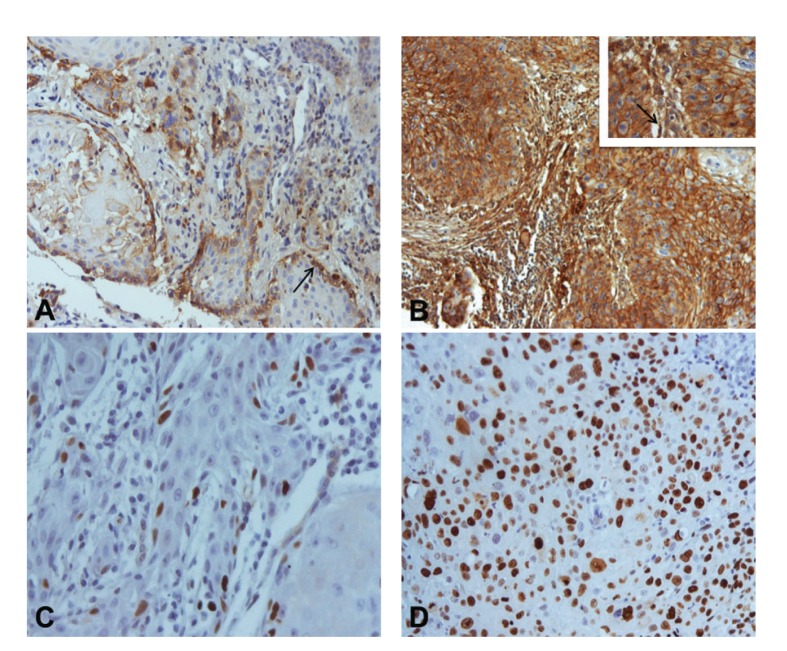
Immunohistochemical expression of EMMPRIN and Ki-67 in oral squamous cell carcinomas: (A) EMMPRIN score 1+ expression with predominantly peripheral distribution pattern (arrow) (×200); (B) EMMPRIN score 3+ expression with staining homogeneously distributed by the tumor islands (×100). Inset: higher magnification (×400). Note peritumoral fibroblast staining (arrow); (C) Ki-67 expression in less than 50% of tumor cells (×400); (D) Ki-67 expression in more than 50% of tumor cells (×400).

Immunohistochemistry was performed in 74 human OSCC tissues to evaluate the extent and patterns of EMMPRIN protein expression. All cases presented membrane staining for EMMPRIN on tumor cells. Additionally, in 61 cases (82.4%), cytoplasmic expression was also observed. On the basis of EMMPRIN immunoexpression, cases were classified as 1+ in 2 (2.7%), 2+ in 16 (21.6%), and 3+ in 56 (75.7%) cases (Figures 1(A) and 1(B)). Staining of this protein was detected predominantly at the periphery of the tumor islands (45; 60.8%) or present homogenously within the tumor islands (29; 39.2%) (Figures 1(A) and 1(B)). We observed also that EMMPRIN expression was seen in peritumoral fibroblasts in 65 (87.8%) cases (Figures 1(A) and 1(B)). Fibroblasts were identified by an experienced pathologist based on its histomorphological features. In cases in which difficulties existed in fibroblast identification, we recurred to coloration for vimentin and smooth muscle actin. Apparently, normal mucosa adjacent to primary tumor presented a strong EMMPRIN staining in basal and suprabasal epithelial layers.

We compared EMMPRIN expression in OSCC tissue samples with patient clinicopathological variables. A positive association of EMMPRIN expression with histological grade was noted where G2/G3 tumors presented EMMPRIN overexpression more often than G1 tumors (*P* = 0.002) (Table 1). Although no significant association was found between EMMPRIN overexpression and clinical stage, when we divided clinical stage into initial stage (I/II) tumors and advanced stage tumors (III/IV), we observed a significant association between EMMPRIN overexpression and advanced tumor stages (*P* = 0.015). No other significant relationship between EMMPRIN expression and the listed clinicopathological parameters was found. Moreover, we did not find significant relationship between EMMPRIN distribution pattern (homogeneously or peripherically), EMMPRIN fibroblast staining, and the listed clinicopathological parameters.

### 3.2. EMMPRIN and Ki-67 Correlation

Ki-67 expression was detected in 72 cases (97.3%). Thirty-six cases (48.6%) were classified as low proliferative tumors and 38 (51.4%) as high proliferative tumors (Figures 1(C) and 1(D)). The intensity of the marker was similar and homogeneous in almost all cases. The only association between Ki-67 and the clinicopathological variables was observed with histological grade (*P* = 0.009) (Table 1).

EMMPRIN was positively correlated with Ki-67 expression (*ρ* = 0.33; *P* = 0.004). Tumors with EMMPRIN overexpression (*n* = 56) presented high Ki-67 expression in 64.7% (*n* = 34) of the cases. By contrast, only 22.2% (4/18) of tumors without EMMPRIN overexpression expressed high levels of Ki-67 protein.

### 3.3. Survival Analysis

The mean follow-up for all patients was 36.45 ± 31.7 months and mean follow-up for living patients was 52.05 ± 33.02 months. At the end of our study, 39 patients (52.7%) were alive without oral cancer, one patient (1.4%) was alive with oral cancer, 33 (44.6%) had died as a result of the oral cancer, and one patient (1.4%) had died as a result of cardiovascular disease. The cumulative 3-year cancer-specific survival (CSS) rate was 55.8% and recurrence-free survival (RFS) was 46.6%.

**Figure 2:**
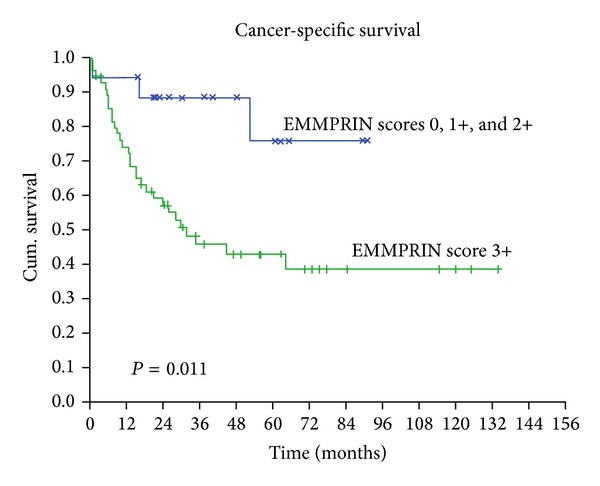
Kaplan-Meier curves according to EMMPRIN expression demonstrating a worse cancer-specific survival in the OSCC patients with overexpression of this protein.

On a univariate analysis using the Kaplan-Meier method and log-rank test, we measured the influence of the clinical-pathological and immunoexpression variables on the survival of patients with OSCC. EMMPRIN overexpression was statistically associated with a worse CSS (*P* = 0.011) (Figure 2; Table 2). Among the clinicopathological characteristics, tumor size (*P* < 0.001), *N* status (*P* = 0.003), tumor stage (*P* < 0.001), treatment modality (*P* = 0.05), and histological grade (*P* = 0.037) were also statistically associated with a worse CSS (Table 2). As also described in Table 2, we observed a significant association between RFS and gender (*P* = 0.013), tumor size (*P* = 0.009), *N* status (*P* = 0.006), tumor stage (*P* = 0.009), margin status (*P* = 0.003), and perineural permeation (*P* = 0.041).

In the multivariate analyses using Cox regression method, we found an association of EMMPRIN overexpression with poor survival (*P* = 0.034; Table 3), thus revealing EMMPRIN overexpression as an adverse independent prognostic factor for CSS in OSCC. In RFS, gender (*P* = 0.030) and margin status (*P* = 0.019) reveal an independent prognostic value for these tumors (Table 4).

## 4. Discussion

Recent studies have reported the biological and clinical role of EMMPRIN receptor in several cancers in the last decades [4, 5, 24]. However, the influence of this receptor in OSCC is poorly understood. In the present study, we aimed to evaluate the expression of EMMPRIN protein in OSCC and to analyse the correlation of this receptor with clinicopathological characteristics, tumor proliferation, and patient's outcome.

Our study showed that EMMPRIN protein was present in all OSCC cases and overexpressed in more than two-thirds of the cases. This result is in accordance with the notably high expression of this glycoprotein in squamous cell carcinomas of head and neck region reported by Riethdorf et al. [5]. By analyzing EMMPRIN expression in multitumor TMAs, they observed expression in more than 95% of squamous cell carcinoma of oral cavity and 100% in squamous cell carcinomas of salivary glands. Lower expression values have been reported by Gou et al. [25] in laryngeal carcinomas (87.5%), Zhu et al. [17] in esophageal squamous cell carcinomas (85%), and Huang et al. [11] in tongue squamous cell carcinomas (67%). They also observed a significantly higher expression on tumor cells than in the noncancerous epithelium. Vigneswaran et al. [26] found a strong EMMPRIN expression in more than 90% of tumor cells in carcinoma in situ and early-invasive OSCC and also a significant higher expression compared with normal oral mucosa. The authors also found an increasing expression of this marker in oral leukoplakias gradually correlated with the degree of dysplasia, suggesting that EMMPRIN overexpression occurs at an early step of oral carcinogenesis and contributes to oral tumorigenesis. These data highlight the potential important role of EMMPRIN in OSCC.

We observed EMMPRIN expression in tumor cell membrane and also in the cytoplasm of some cases, in concordance with other studies [12, 14, 19]. Although our aim was to analyse the expression of EMMPRIN in tumour cells, we detected the presence of this receptor in peritumoral fibroblasts as described by Vigneswaran et al. [26]. Furthermore, EMMPRIN expression showed a predominantly peripheric/basal distribution pattern in the tumor islands in most of our cases. This was reported in other works suggesting a more frequent distribution of this receptor in tumor cells with a more proliferative phenotype [11, 26].

In order to assess the relationship of EMMPRIN with proliferative activity, we evaluated the expression of Ki-67 in these tumors and found a significant correlation between these two proteins. To our knowledge, this correlation has not been reported in OSCC, although Yang et al. [14] described a positive association between EMMPRIN expression and Ki-67 index labelling and also with tumor size in adenoid cystic carcinomas. Similar results were reported by Zheng et al. [27] showing a positive correlation between the two markers in gastric carcinomas. These results are in line with ours, suggesting that EMMPRIN might be relevant for the tumor proliferation and tumor growth of OSCC. Furthermore, knockdown of EMMPRIN in head and neck carcinomas decreased cellular proliferation and tumor growth in vitro and in vivo analyses [28–30]. Mechanisms involved in tumor proliferation via EMMPRIN are poorly understood, but some authors have described the role of this receptor in association with cyclophilin A in the activation of ERK1/2 and p38 pathways [31].

We found that EMMPRIN expression was significantly associated with histological grade. Moderately or poorly differentiated tumors showed more EMMPRIN overexpression than well-differentiated tumors. Zhu et al. [17] observed the same positive association in 86 esophageal squamous cell carcinomas. Clinical stage and tumor size have been positively related with EMMPRIN expression in several cancers including head and neck cancers [11, 12, 25]. We observed that EMMPRIN overexpression was more frequently found in patients with advanced clinical stage (III/IV), emphasizing the biological significance of this marker to tumor growth and progression of OSCC. We did not find any other significant relation with other clinicopathological parameters although association with nodal metastasis was observed by others [32].

The influence of EMMPRIN expression on patient's survival has been reported in glioblastomas, seminomas, and other cancers including tongue, salivary gland, esophageal, ovary, colorectal, breast, bladder, and lung cancers [11–15, 17, 19, 33–36]. In our univariate analysis, we found that cases with EMMPRIN overexpression were associated with a lower CSS (*P* = 0.011) additionally with other clinical variables such as TNM and clinical stage. Nevertheless, in the multivariate analysis for CSS, EMMPRIN protein was the only independent prognostic factor (*P* = 0.034), revealing the adverse independent impact of EMMPRIN overexpression on the survival of patients with OSCC. To our knowledge, this is the first report of the independent prognostic value of EMMPRIN in a cohort of patients with squamous cell carcinoma of the oral cavity. Previously, Huang et al. [11] described the independent significant influence of this receptor in the overall survival of patients with squamous cell carcinomas of the tongue. This could be an important result suggesting the use of this receptor as a prognostic biomarker in OSCC. Interestingly, some studies report that EMMPRIN might be even a predictive marker of chemoresistance in head and neck carcinomas [8]. The influence of this receptor on patient's prognostic could be related to the multiple biological functions of this protein on tumor cells such as proliferation, migration, invasion, angiogenesis, and dissemination on OSCC [4]. Studies have described the role of EMMPRIN in the stimulation of several metalloproteinases and proangionenic factors from tumor and adjacent stroma cells that could contribute to tumor multistep pathogenesis [27]. It would be interesting to analyse the relationship of EMMPRIN expression and molecules involved in different pathways, such as EGFR, MMP's, and VEGFR's, in a larger sample of OSCC.

The understanding of the different pathways involved in oral tumorigenesis could reveal new candidate target molecules for anticancer drugs. Anti-EGFR targeted therapies are currently available for head and neck cancer but with modest results [37]. New anticancer therapies directed to molecular targets on oral cancer cells are needed and some molecules have been proposed, including EMMPRIN receptor [5, 9, 11, 38]. The high expression of this receptor in OSCC, the cell membrane location, the biological role on tumor growth, invasion, dissemination, and the influence in the patient's prognosis make EMMPRIN a strong candidate for a potential molecular target for monoclonal therapies against this receptor in OSCC. Anti-EMMPRIN molecular therapies showed growth inhibitory effect on head and neck squamous cell carcinoma, alone and in combination with radiotherapy in vitro and in vivo [39]. Sweeny et al. [40] reported a promising extracellular drug conjugate (EDC22), capable of inhibiting HNSCC cell proliferation in vitro and in vivo, with better results than with radiation or cisplatin monotherapy.

In conclusion, our results reveal that EMMPRIN protein is frequently overexpressed in OSCC, especially in high proliferative tumors, suggesting that it might be involved in the growth of these tumors. The independent value of EMMPRIN overexpression in CSS indicates that this protein could be used as an important biological prognostic marker to identify high risk OSCC patients, helping in making the right decision as to the appropriate treatment. Furthermore, the high expression of this receptor could be regarded as potential therapeutic target against OSCC.

## Figures and Tables

**Table 1:** Clinicopathological characteristics of the 74 patients with OSCC and their association with EMMPRIN and Ki-67 expressions.

Factor	*N* (%)	EMMPRIN overexpression		Ki-67 high expression	
		*N* (%)	*P* value	*N* (%)	*P* value
All cases	74 (100%)	56 (75.7%)		38 (51.4%)	
Gender					
Female	19 (25.7%)	11 (63.2%)	0.140	8 (42.1%)	0.350
Male	55 (74.3%)	44 (80%)		30 (54.5%)	
Age					
<62 years	37 (50%)	30 (81.1%)	0.278	22 (59.5%)	0.163
≥62 years	37 (50%)	26 (70.3%)		16 (43.2%)	
Location					
Labial mucosa	7 (9.5%)	6 (85.7%)	0.430	1 (14.3%)	0.396
Floor of the mouth	10 (13.5%)	7 (70%)		8 (80%)	
Tongue	24 (32.4%)	15 (62.5%)		13 (54.2%)	
Buccal mucosa	5 (6.8%)	5 (100%)		2 (40%)	
Retromolar trigone	11 (14.9%)	8 (72.7%)		5 (45.5%)	
Hard palate	9 (12.2%)	8 (88.9%)		3 (55.6%)	
Gingiva	8 (10.8%)	7 (87.5%)		4 (50%)	
Tumor size					
T1	13 (17.6%)	8 (61.5%)	0.132	6 (46.2%)	0.396
T2	29 (39.2%)	20 (69%)		12 (41.4%)	
T3	9 (12.2%)	9 (100%)		6 (66.7%)	
T4	23 (31%)	19 (82.6%)		14 (60.9%)	
N status					
N0	41 (55.4%)	28 (68.3%)	0.349	16 (39%)	0.052
N1	12 (16.2%)	11 (91.7%)		7 (58.3%)	
N2	17 (23%)	14 (82.4%)		11 (64.7%)	
N3	4 (5.4%)	3 (75%)		4 (100%)	
Stage					
I	12 (16.2%)	8 (66.7%)	0.064	6 (50%)	0.080
II	23 (31.1%)	14 (60.9%)		7 (30.4%)	
III	10 (13.5%)	10 (100%)		7 (70%)	
IV	29 (39.2%)	24 (82.8%)		18 (62.1%)	
Treatment modality					
SG	28 (37.8%)	20 (71.4%)	0.633	12 (42.9%)	0.522
SG + RT	23 (31.3%)	17 (73.9%)		13 (56.5%)	
CT + SG or RCT	23 (31.3%)	19 (82.6%)		13 (56.5%)	
Tumor grade					
G1	42 (56.8%)	26 (61.9%)	0.002	16 (38.1%)	0.009
G2 + G3	32 (43.2%)	30 (93.8%)		22 (68.8%)	
Margin status*					
Free of tumor	33 (57.9%)	23 (69.7%)	0.423	14 (42.4%)	0.236
With tumor	24 (42.1%)	19 (79.2%)		14 (58.3%)	
Perineural permeation					
Absent	66 (89.2%)	50 (75.8%)	0.962	33 (50%)	0.504
Present	8 (10.8%)	6 (75%)		5 (62.5%)	
Lymphatic invasion					
Absent	58 (78.4%)	46 (79.3%)	0.165	30 (51.7%)	0.903
Present	16 (21.6%)	10 (62.5%)		8 (50%)	

SG: surgery; RT: radiotherapy; CT: chemotherapy; RCT: radiochemotherapy.

*Not determined in the 17 cases.

**Table 2:** Univariable analysis of cancer-specific and recurrence-free survivals (at 3-years of follow-up).

Factor	*N*	Dead	Cancer-specific survival	*P* value	*N*	Recurrence	Recurrence-free survival	*P* value
Gender								
Female	19	10	52.1	0.265	15	11	33.3	0.013
Male	55	23	57.8		47	20	50.7	
Age								
<62 yrs	37	19	46.4	0.324	29	16	37.7	0.546
≥62 yrs	37	14	65.2		33	15	54.1	
Location								
Lip	7	1	83.3	0.491	7	1	83.3	0.383
Tongue	10	5	70		10	8	20	
Floor of the mouth	24	10	61.3		20	8	63.3	
Gingiva	5	2	60		4	2	50	
Retromolar trigone	11	6	30.7		8	4	37.5	
Hard palate	9	4	53.3		7	4	42.9	
Buccal mucosa	8	5	37.5		6	4	33.3	
Tumor size								
T1	13	1	88.9	<0.001	13	4	83.3	0.009
T2	29	9	70.4		28	12	53.4	
T3	9	5	30.5		8	5	30	
T4	23	18	16.9		13	10	23.1	
N status								
0	41	11	76.9	0.003	37	13	63.5	0.006
1	12	7	17.8		10	7	18	
2	17	13	24.2		13	10	15.4	
3	4	2	50		2	1	50	
Stage								
I	12	1	88.9	<0.001	12	4	71.6	0.009
II	23	6	77.4		22	8	60	
III	10	5	33.3		9	5	34.6	
IV	29	21	26.9		19	14	23.7	
Tumor grade								
G1	42	13	67.8	0.037	36	15	55.7	0.195
G2/G3	32	20	42.1		26	16	35.3	
Treatment modality								
SG	28	28	76.2	0.050	26	10	55.2	0.134
SG + RT	23	23	47.7		21	13	32.8	
CT + SG or RCT	23	23	37.8		15	8	50	
Margin status*								
Free of tumor	33	9	79.7	0.157	31	9	68.4	0.003
With tumor	24	11	50.1		23	16	26.1	
Perineural permeation								
Absent	66	28	58.2	0.243	54	25	49.8	0.041
Present	8	5	37.5		8	6	25	
Lymphatic invasion								
Absent	58	27	55.1	0.849	47	24	46.5	0.824
Present	16	6	61.1		15	7	53.3	
EMMPRIN expression								
0, 1+, 2+	18	3	88.5	0.011	17	8	58.8	0.882
3+ (overexpression)	58	30	45.7		45	23	43.3	
EMMPRIN distribution								
Homogeneous	45	18	59.2	0.503	37	18	50.5	0.898
Heterogeneous (periphery)	29	15	50.7		25	13	39.6	
EMMPRIN fibroblasts								
Absent	9	6	20.8	0.092	8	6	18.8	0.088
Present	65	27	60.1		54	25	50.6	
Ki-67								
<50% (low expression)	36	12	69.4	0.111	32	14	56.2	0.560
≥50% (high expression)	38	11	45		30	17	39.7	

SG: surgery; RT: radiotherapy; CT: chemotherapy; RCT: radiochemotherapy.

*Not determined in the 17 cases.

**Table 3:** Multivariable analysis of cancer-specific survival on variables with significant effect in univariable analysis.

Variable^a^	*P* value	HR	95% CI
Stage	0.205	1.640	0.763–3.524
T status	0.075	1.824	0.941–3.532
N status	0.353	0.799	0.497–1.283
Treatment modality	0.673	1.110	0.685–1.797
Tumor grade	0.411	1.405	0.624–3.160
EMMPRIN expression	0.034	3.894	1.106–13.709

HR: hazard ratio; CI: confidence interval for HR.

^a^Variables included in multivariable Cox regression analysis using enter method; stage (ordinal variable); T status (ordinal variable); N status (ordinal variable); treatment modality (ordinal variable); tumor grade, G2 + G3 versus G1 (reference category); EMMPRIN expression, positive versus negative (reference category).

**Table 4:** Multivariable analysis of recurrence-free survival on variables with significant effect in univariable analysis.

Variable^a^	*P* value	HR	95% CI
Gender	0.030	2.849	1.110–7.315
Stage	0.384	1.589	0.560–4.512
T status	0.365	1.372	0.693–2.718
N status	0.700	0.836	0.337–2.077
Margin status	0.019	3.081	1.205–7.879
Perineural permeation	0.498	1.454	0.492–4.294

HR: hazard ratio; CI: confidence interval for HR.

^a^Variables included in multivariable Cox regression analysis using enter method; gender, female versus male (reference category); stage (ordinal variable); T status (ordinal variable); N status (ordinal variable); margin status, with tumor versus without tumor (reference category); perineural permeation, present versus absent (reference category).

## References

[1] Warnakulasuriya S (2009). Global epidemiology of oral and oropharyngeal cancer. *Oral Oncology*.

[2] Jemal A, Bray F, Center MM, Ferlay J, Ward E, Forman D (2011). Global cancer statistics. *CA Cancer Journal for Clinicians*.

[3] Hahn WC, Weinberg RA (2002). Rules for making human tumor cells. *The New England Journal of Medicine*.

[4] Iacono KT, Brown AL, Greene MI, Saouaf SJ (2007). CD147 immunoglobulin superfamily receptor function and role in pathology. *Experimental and Molecular Pathology*.

[5] Riethdorf S, Reimers N, Assmann V (2006). High incidence of EMMPRIN expression in human tumors. *International Journal of Cancer*.

[6] Zucker S, Hymowitz M, Rollo EE (2001). Tumorigenic potential of extracellular matrix metalloproteinase inducer. *The American Journal of Pathology*.

[7] Tang Y, Nakada MT, Kesavan P (2005). Extracellular matrix metalloproteinase inducer stimulates tumor angiogenesis by elevating vascular endothelial cell growth factor and matrix metalloproteinases. *Cancer Research*.

[8] Huang Z, Wang L, Wang Y (2013). Overexpression of CD147 contributes to the chemoresistance of head and neck squamous cell carcinoma cells. *Journal of Oral Pathology & Medicine*.

[9] Suzuki S, Ishikawa K (2014). Combined inhibition of EMMPRIN and epidermal growth factor receptor prevents the growth and migration of head and neck squamous cell carcinoma cells. *International Journal of Oncology*.

[10] Afonso J, Longatto-Filho A, Baltazar F (2011). CD147 overexpression allows an accurate discrimination of bladder cancer patients’ prognosis. *European Journal of Surgical Oncology*.

[11] Huang Z, Huang H, Li H, Chen W, Pan C (2009). EMMPRIN expression in tongue squamous cell carcinoma. *Journal of Oral Pathology and Medicine*.

[12] Piao S, Zhao S, Guo F (2012). Increased expression of CD147 and MMP-9 is correlated with poor prognosis of salivary duct carcinoma. *Journal of Cancer Research and Clinical Oncology*.

[13] Stenzinger A, Wittschieber D, von Winterfeld M (2012). High extracellular matrix metalloproteinase inducer/CD147 expression is strongly and independently associated with poor prognosis in colorectal cancer. *Human Pathology*.

[14] Yang X, Dai J, Li T (2010). Expression of EMMPRIN in adenoid cystic carcinoma of salivary glands: correlation with tumor progression and patients’ prognosis. *Oral Oncology*.

[15] Zhao S, Ma W, Zhang M (2013). High expression of CD147 and MMP-9 is correlated with poor prognosis of triple-negative breast cancer (TNBC) patients. *Medical Oncology *.

[16] Zhu S, Chu D, Zhang Y (2013). EMMPRIN/CD147 expression is associated with disease-free survival of patients with colorectal cancer. *Medical Oncology*.

[17] Zhu S, Li Y, Mi L (2011). Clinical impact of HAb18G/CD147 expression in esophageal squamous cell carcinoma. *Digestive Diseases and Sciences*.

[18] Yang M, Yuan Y, Zhang H (2013). Prognostic significance of CD147 in patients with glioblastoma. *Journal of Neuro-Oncology*.

[19] Xu XY, Lin N, Li YM (2013). Expression of HAb18G/CD147 and its localization correlate with the progression and poor prognosis of non-small cell lung cancer. *Pathology—Research and Practice*.

[20] Brandwein-Gensler M, Smith RV (2010). Prognostic indicators in head and neck oncology including the new 7th edition of the AJCC staging system. *Head and Neck Pathology*.

[21] Barnes L, Eveson JW, Reichart P, Sidransky D (2005). *Pathology and Genetics of Head and Neck Tumours*.

[22] Monteiro LS, Diniz-Freitas M, Garcia-Caballero T, Forteza J, Fraga M (2010). EGFR and Ki-67 expression in oral squamous cell carcinoma using tissue microarray technology. *Journal of Oral Pathology & Medicine*.

[23] Carlos de Vicente J, Herrero-Zapatero A, Fresno MF, López-Arranz JS (2002). Expression of cyclin D1 and Ki-67 in squamous cell carcinoma of the oral cavity: clinicopathological and prognostic significance. *Oral Oncology*.

[24] Li Y, Xu J, Chen L (2009). HAb18G (CD147), a cancer-associated biomarker and its role in cancer detection. *Histopathology*.

[25] Gou X, Chen H, Jin F (2014). Expressions of CD147, MMP-2 and MMP-9 in laryngeal carcinoma and its correlation with poor prognosis. *Pathology & Oncology Research*.

[26] Vigneswaran N, Beckers S, Waigel S (2006). Increased EMMPRIN (CD 147) expression during oral carcinogenesis. *Experimental and Molecular Pathology*.

[27] Zheng HC, Takahashi H, Murai Y (2006). Upregulated EMMPRIN/CD147 might contribute to growth and angiogenesis of gastric carcinoma: a good marker for local invasion and prognosis. *The British Journal of Cancer*.

[28] Sweeny L, Liu Z, Bush BD (2012). CD147 and AGR2 expression promote cellular proliferation and metastasis of head and neck squamous cell carcinoma. *Experimental Cell Research*.

[29] Yang X, Zhang P, Ma Q (2012). EMMPRIN silencing inhibits proliferation and perineural invasion of human salivary adenoid cystic carcinoma cells *in vitro* and *in vivo*. *Cancer Biology & Therapy*.

[30] Zhu C, Pan Y, He B (2011). Inhibition of CD147 gene expression via RNA interference reduces tumor cell invasion, tumorigenicity and increases chemosensitivity to cisplatin in laryngeal carcinoma Hep2 cells. *Oncology Reports*.

[31] Li M, Zhai Q, Bharadwaj U (2006). Cyclophilin A is overexpressed in human pancreatic cancer cells and stimulates cell proliferation through CD147. *Cancer*.

[32] Huang C, Sun Z, Sun Y (2012). Association of increased ligand cyclophilin A and receptor CD147 with hypoxia, angiogenesis, metastasis and prognosis of tongue squamous cell carcinoma. *Histopathology*.

[33] Pinheiro C, Longatto-Filho A, Simões K (2009). The prognostic value of CD147/EMMPRIN is associated with monocarboxylate transporter 1 co-expression in gastric cancer. *European Journal of Cancer*.

[34] Davidson B, Goldberg I, Berner A, Kristensen GB, Reich R (2003). EMMPRIN (extracellular matrix metalloproteinase inducer) is a novel marker of poor outcome in serous ovarian carcinoma. *Clinical and Experimental Metastasis*.

[35] Xue YJ, Lu Q, Sun ZX (2011). CD147 overexpression is a prognostic factor and a potential therapeutic target in bladder cancer. *Medical Oncology*.

[36] Bi XC, Liu JM, He HC (2012). Extracellular matrix metalloproteinase inducer: a novel poor prognostic marker for human seminomas. *Clinical and Translational Oncology*.

[37] Rabinowits G, Haddad RI (2012). Overcoming resistance to EGFR inhibitor in head and neck cancer: a review of the literature. *Oral Oncology*.

[38] Monteiro LS, Delgado ML, Ricardo S (2013). Phosphorylated mammalian target of rapamycin is associated with an adverse outcome in oral squamous cell carcinoma. *Oral Surgery, Oral Medicine, Oral Pathology, Oral Radiology*.

[39] Dean NR, Newman JR, Helman EE (2009). Anti-EMMPRIN monoclonal antibody as a novel agent for therapy of head and neck cancer. *Clinical Cancer Research*.

[40] Sweeny L, Hartman YE, Zinn KR (2013). A novel extracellular drug conjugate significantly inhibits head and neck squamous cell carcinoma. *Oral Oncology*.

